# Enhancing Photocatalytic Hydrogen Production of g-C_3_N_4_ by Selective Deposition of Pt Cocatalyst

**DOI:** 10.3390/nano11123266

**Published:** 2021-11-30

**Authors:** Yang Li, Yue Lu, Zhaoyu Ma, Lianqing Dong, Xiaofang Jia, Junying Zhang

**Affiliations:** 1 School of Physics, Beihang University, Beijing 100191, China; liyang1619125@buaa.edu.cn (Y.L.); Luyue@buaa.edu.cn (Y.L.); 18338208022@163.com (Z.M.); jiaxiaofang@buaa.edu.cn (X.J.); 2 Beijing Institute of Space Mechanics & Electricity, Beijing 100094, China; XPHDLQ@163.com

**Keywords:** graphitic carbon nitride, platinum, selective deposition, photocatalytic hydrogen production

## Abstract

Graphitic carbon nitride (g-C_3_N_4_) has been widely studied as a photocatalyst for the splitting of water to produce hydrogen. In order to solve the problems of limited number of active sites and serious recombination rate of charge-carriers, noble metals are needed as cocatalysts. Here, we selectively anchored Pt nanoparticles (NPs) to specific nitrogen species on the surface of g-C_3_N_4_ via heat treatment in argon–hydrogen gas mixture, thus achieving g-C_3_N_4_ photocatalyst anchored by highly dispersed homogeneous Pt NPs with the co-existed metallic Pt^0^ and Pt^2^^+^ species. The synergistic effect of highly dispersed metallic Pt^0^ and Pt^2^^+^ species makes the catalyst exhibit excellent photocatalytic performance. Under the full-spectrum solar light irradiation, the photocatalytic hydrogen production rate of the photocatalyst is up to 18.67 mmol·g^−1^·h^−1^, which is 5.1 times of the catalyst prepared by non-selective deposition of Pt NPs.

## 1. Introduction

With the development of world economy and the increase of population, the demand for energy is increasing continually [1,2]. In order to reduce the dependence on non-renewable energy, it is very important to develop environmentally friendly alternative energy. In 1972, Fujishima and Honda creatively revealed the possibility for photocatalytic water splitting to generate hydrogen by TiO_2_ photoelectrode [3]. Since then, investigating on the semiconductor photocatalyst have been carried out extensively [4,5,6,7,8,9,10,11]. Among the photocatalytic semiconductors studied, g-C_3_N_4_ has been considered as an ideal photocatalyst with moderate band gap (2.7 eV) and stable physical and chemical properties [12,13,14,15,16,17,18,19].

While g-C_3_N_4_ has the aforementioned superiority, the feature of low electrical conductivity and high recombination rate of charge-carriers limit its application [20,21,22,23]. To overcome these two barriers, noble metals are often used as cocatalysts to accelerate electron transfer to inhibit recombination and reduce redox overpotential at the active sites [24,25]. Nevertheless, noble metals are relatively expensive, so it is of importance to explore a more efficient method for dispersion of noble metals and their combination with g-C_3_N_4_ [26,27]. To pursue this goal, a large number of studies have been reported on the dispersion and binding of noble metals on g-C_3_N_4_ as cocatalysts [28,29,30,31]. Among them, Zhu et al. [31] found that the anchoring environment of noble metals on carbon nitride could be adjusted through chemical treatment. Wang et al. proposed that different types of nitrogen play different roles in bonding noble metals, which was confirmed by experiments and calculations. Among the nitrogen species involved, tri-coordination graphitized nitrogen (N-(C)_3_) is considered to be beneficial to the nucleation and dispersion of noble metal NPs, while pyridine nitrogen (C-N=C) may play a role in the anchoring and dispersion of noble metal NPs [32]. This enlightens that we can selectively deposit Pt NPs on g-C_3_N_4_ by tuning the interaction of Pt with the different chemically-boned nitrogen atoms, which will greatly influence the photocatalytic activity of g-C_3_N_4_.

In the present work, three deposition methods of Pt NPs were conducted based on the lattice sites of N atoms. For the first two conditions, Pt NPs were selectively deposited on N-(C)_3_ and C-N=C of g-C_3_N_4_ by heat treatment in argon–hydrogen mixture and air, respectively. For the last case, Pt NPs were indiscriminately deposited on g-C_3_N_4_ by photoreduction method. The photocatalytic hydrogen production test indicated that the catalysts obtained by selectively depositing Pt on the N-(C)_3_ site of g-C_3_N_4_ (named CNH-Pt) showed best photocatalytic performance. Under full-spectrum solar light irradiation, the photocatalytic hydrogen production rate of CNH-Pt is 622.3 and 5.1 times those of the pure g-C_3_N_4_ and the catalyst with undifferentiated Pt NPs deposition (CNP-Pt), respectively.

## 2. Materials and Methods

### 2.1. Materials

Urea (A.R. > 99%) and triethanolamine (A.R.) were purchased from Shanghai Aladdin Bio-Chem Technology Co., Ltd., Shanghai, China, and chloroplatinic acid hexahydrate (A.R. Pt ≥ 37.5%) was purchased from Shanghai Macklin Biochemical Co., Ltd., Shanghai, China. All reagents were used without further purification.

### 2.2. Preparation of Catalyst

Preparation of g-C_3_N_4_: g-C_3_N_4_ was synthesized according to the method reported in previous literature [33]. 10 g Urea was heated at 500 °C for 2 h in a muffle furnace with a ramp rate of 5 °C min^−1^, then cooled naturally and ground to get milky powder.

Preparation of Pt-loaded g-C_3_N_4_:

Preparation of CNH-Pt (Pt NPs are selectively deposited on N-(C)_3_): 300 mg g-C_3_N_4_ powder was dispersed in 20 mL deionized water and the solution was ultrasonically vibrated for 30 min. Then appropriate amount of H_2_PtCl_6_·6H_2_O solution was added drop by drop and stirred for 30 min. Finally, the pale-yellow powder was obtained by freeze drying. 100 mg of dried pale-yellow powder was heated in a tubular furnace at 400 °C for 1 h in argon–hydrogen (90 vol%Ar-10 vol% H_2_) atmosphere to obtain grey powder. The sample with 3 wt% Pt loading was named as CNH-Pt.

Preparation of CNA-Pt (Pt NPs are selectively deposited on C-N=C): Under the same conditions, the aforementioned pale-yellow powder was annealed in air atmosphere instead of argon–hydrogen atmosphere, and the sample with 3 wt% Pt loading was named as CNA-Pt.

Preparation of CNP-Pt (Pt NPs are deposited indiscriminately): Pt NPs were indiscriminately loaded onto g-C_3_N_4_ through photoreduction. One hundred milligrams of g-C_3_N_4_ was dispersed in 40 mL water and the solution was ultrasonically vibrated for 30 min. Then a certain amount of H_2_PtCl_6_·6H_2_O solution with the designed nominal 3 wt% Pt was added, and the solution was stirred under the irradiation of a xenon lamp (300 W) for 1 h. Finally, the catalyst was obtained after freeze drying and named as CNP-Pt.

As the control group, the sample obtained by annealing of g-C_3_N_4_ in argon–hydrogen atmosphere without Pt was named as CNH, and the sample obtained by annealing of g-C_3_N_4_ in air without Pt was named as CNA. The annealing time, temperature and heating rate were consistent with CNH-Pt.

First-principles calculation methods and characterization and photocatalytic tests of the materials are detailed in Supporting Information.

## 3. Results and Discussion

**Figure 1:**
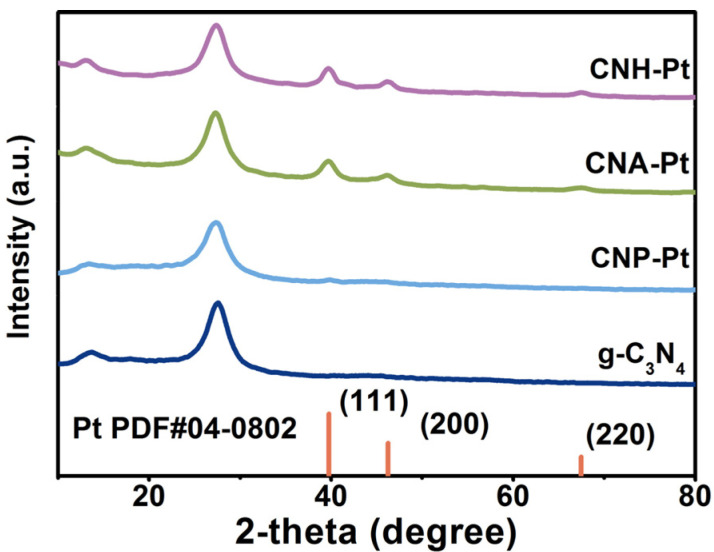
XRD patterns of the catalysts.

The crystal structures of the samples can be depicted through the X-ray powder diffraction (XRD) pattern, as shown in Figure 1. Pure g-C_3_N_4_ shows two characteristic peaks located at around 13.2° and 27.4°, which are attributed to the in-plane ordering of tri-s-triazine units (indexed as (100)) and the interlayer stacking of conjugated aromatic systems (indexed as (002)), respectively [34]. CNH and CNA also show similar diffraction patterns, indicating that high temperature heat treatment does not destroy the crystal structure of g-C_3_N_4_ (Figure S1). The diffraction peaks at around 13.2° and 27.4° also appear in the patterns of samples CNH-Pt, CNA-Pt, and CNP-Pt, indicating that g-C_3_N_4_ preserve its crystal structure with Pt NPs loaded into it. In addition, weak peaks at 39.7° have been detected in CNH-Pt, CNA-Pt, and CNP-Pt, which are diffraction peaks corresponding to Pt (111) crystal plane [31], which can be concluded that Pt NPs have been loaded onto g-C_3_N_4_ by three different methods. Among them, the diffraction peaks of CNH-Pt and CNA-Pt at 39.7° are obvious, which is due to the good crystallinity of Pt obtained by high temperature heat treatment. The diffraction peak of CNP-Pt at 39.7° is weaker, which may be because the photoreduction process is carried out at 10 °C, and the lower temperature makes part of Pt exist in amorphous form and has not been detected. In addition, inductively coupled plasma optical emission spectrometer (ICP-OES) results show that the actual Pt loads concentration of CNH-Pt, CNA-Pt, and CNP-Pt are all about 3 wt% (Table S1).

**Figure 2:**
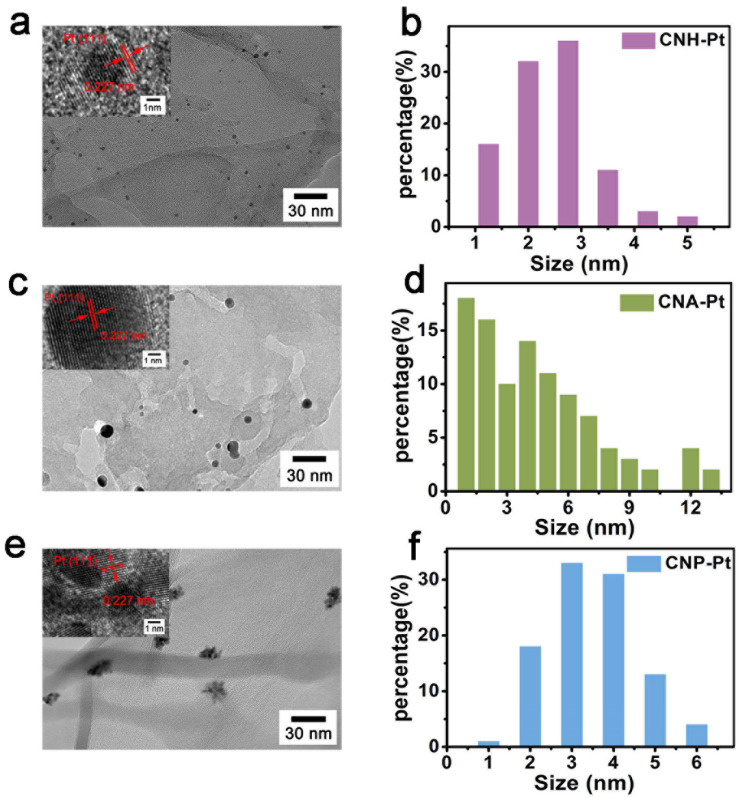
TEM images and Pt NPs size distribution of CNH-Pt (**a**,**b**), CNA-Pt (**c**,**d**), and CNP-Pt (**e**,**f**). Insets show HRTEM images of CNH-Pt (**a**), CNA-Pt (**c**), and CNP-Pt (**e**).

Transmission electron microscopy (TEM) images (Figure 2) show the microscopic morphology of g-C_3_N_4_ loaded with Pt NPs. As can be seen from Figure 2a,b, Pt NPs loaded on CNH-Pt have uniform sizes of about 2.5 nm and disperse evenly on the surface of g-C_3_N_4_. However, the size of Pt NPs on CNA-Pt varies from 1 nm to 13 nm (Figure 2c,d). The size of Pt NPs on CNP-Pt is similar to that on CNH-Pt (about 3.5 nm), but multiple Pt NPs accumulate into clusters, and each Pt particle does not exist independently (Figure 2e,f). HRTEM images of CNH-Pt, CNA-Pt, and CNP-Pt are shown in the insets of 2a, 2c and 2e, respectively. The Pt NPs lattice distances of CNH-Pt, CNA-Pt, and CNP-Pt are identical at 0.227 nm, corresponding to the (111) plane of metallic Pt [35], presenting a consistency with the XRD results. Meanwhile, it can be observed from the illustration of Figure 2e that some Pt NPs have poor crystallinity, which is consistent with the weak diffraction peak of CNP-Pt at 39.7° in the XRD pattern (Figure 1).

**Figure 3:**
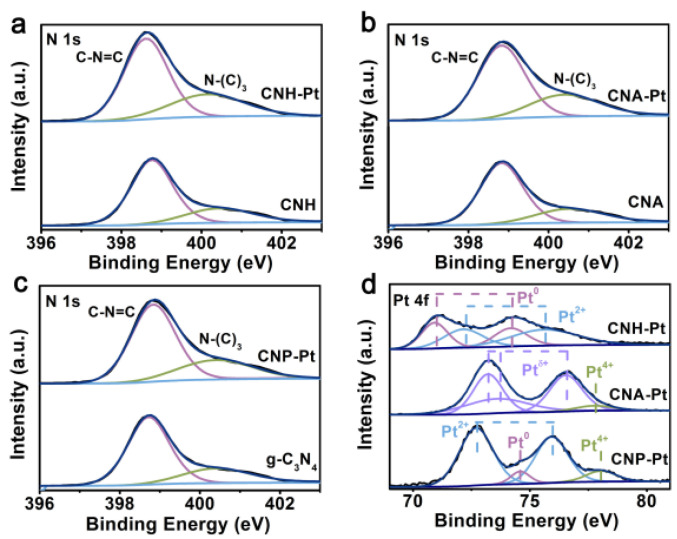
XPS spectra of N 1s of CNH-Pt and CNH (**a**), CNA-Pt and CNA (**b**), CNP-Pt, and g-C_3_N_4_ (**c**). (**d**) Pt 4f spectra of CNH-Pt, CNA-Pt, and CNP-Pt.

X-ray photoelectron spectroscopy (XPS) analysis was carried out to further study the binding of Pt NPs to g-C_3_N_4_. The N 1s spectrum is fitted with two peaks located at 398.7 eV and 400.4 eV (Figure 3a–c). A dominant peak at 398.7 eV corresponds to the N_2C_ in C-N=C groups [36,37], while weak peak at the high binding energy 400.4 eV is attributed to the N_3C_ in N-(C)_3_ groups [38]. It is worth noting that the ideal g-C_3_N_4_ has only two functional groups, C-N=C and N-(C)_3_, but the experimentally synthesized g-C_3_N_4_ often contains a small number of C-N-H functional groups. It can be seen from the fitting XPS of the three functional groups (Figure S2) that the content of C-N-H is very small. The C 1s spectrum can be divided into three peaks located at 284.8, 286.6, and 288.2 eV, which are assigned to the adventitious carbon, sp^3^ hybridized carbon (C-(N)_3_) and N–C=N coordination in the framework of g-C_3_N_4_, respectively (Figure S3) [39,40]. After loading Pt NPs, the N 1s peak and C 1s peak of the samples do not change significantly, indicating that the loading of Pt NPs on g-C_3_N_4_ do not destroy its basic structure.

The Pt 4f spectra of CNH-Pt, CNA-Pt and CNP-Pt are shown in Figure 3d. The Pt 4f peak of CNH-Pt can be decomposed into four peaks, and the peaks located at 70.9 eV and 74.2 eV are attributed to metal Pt^0^, and peaks located at 72.2 eV and 75.7 eV are corresponding to Pt^2+^. Metal Pt^0^ can promote electron transport and separation, and Pt^2+^ species can suppress the hydrogen back reaction [41,42]. The coexistence of Pt^0^ and Pt^2+^ makes CNH-Pt possess better photocatalytic performance. The Pt 4f peaks of CNA-Pt can be divided to obtain the peaks of Pt^δ+^ (2 < δ < 4) at 73.2 eV, 73.6 eV, and 76.5 eV, and a small amount of Pt^4+^ at 77.8 eV [37]. Owing to the reducing ability of air is too weak, Pt^4+^ in CNA-Pt can only be reduced to Pt^δ+^ even at high temperature. Similarly, the reducing ability of light is not strong enough to reduce all Pt^4+^ to a lower valence state, resulting in the existence of part of Pt^4+^ in CNP-Pt. The photocatalytic performance of CNA-Pt and CNP-Pt is mediocre due to the incomplete reduction of Pt^4+^ and the absence or small amount of metal Pt^0^. Simultaneously, there are also Pt^2+^ peaks located at 72.7 eV and 75.9 eV and Pt^0^ peaks located at 74.6 eV in CNP-Pt [41,42]. Comparing with CNP-Pt, the binding energy of Pt^2+^ and Pt^0^ peaks of CNH-Pt is lower, indicating that Pt NPs in CNH-Pt can attract more electrons [31].

**Figure 4:**
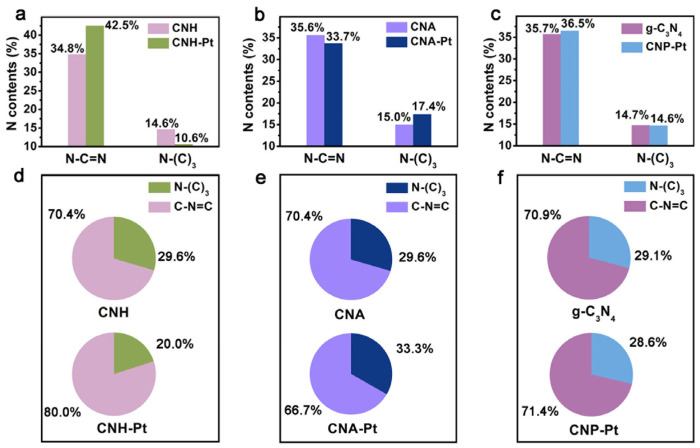
The surface N species content of CNH-Pt and CNH (**a**), CNA-Pt and CNA (**b**), CNP-Pt and g-C_3_N_4_ (**c**). The ratio of N-(C)_3_ and C-N=C before and after Pt loading of CNH (**d**), CNA (**e**), and g-C_3_N_4_ (**f**).

The surface contents of the two N species in different catalysts are depicted in Figure 4a–c, showing no significance difference between CNH, CNA and pure g-C_3_N_4_. However, after loading Pt NPs with three different methods, the surface content of the two N species alters significantly, which is caused by Pt NPs selective anchoring on the different N species [31]. Figure 4d–f shows the variation in the proportions of the two N species on the catalyst surface before and after loading Pt NPs. Compared with CNH, the ratio of N-(C)_3_ in CNH-Pt decrease from 29.6% to 20.0%, indicating that Pt NPs in CNH-Pt are mainly anchored on N-(C)_3_. N-(C)_3_ is believed to play an important role in the nucleation and dispersion of noble metal particles, and the Pt particles bound to it have high adsorption energy [32,43]. Therefore, it is less likely to attract a large number of Pt atoms to form large particles, which also explains the high dispersity and relatively small size of Pt NPs in CNH-Pt. The C-N=C decrease from 70.4% in CNA to 66.7% in CNA-Pt, indicating that Pt NPs in CNA-Pt are mainly supported by C-N=C. C-N=C can be used as an anchor point, and the Pt attached to it has a lower adsorption energy [32,43]. It is easier for Pt atoms to coalesce into larger particles, which is consistent with TEM observations of larger Pt NPs in CNA-Pt (Figure 2c). However, comparing with pure g-C_3_N_4_, the proportion of N species in CNP-Pt is basically maintained. Pt NPs in CNP-Pt are not selectively connected with N species, resulting in a large number of particles agglomerated together without being dispersed. From the fitting results of the N 1s spectra using three functional groups, the change of Pt deposition to ratio of N-(C)_3_ and C-N=C follows the same trend with the results using two functional groups, and the C-N-H content hardly changes before and after loading Pt (Figure S4). Therefore, only C-N=C and N-(C)_3_ will be discussed below.

**Figure 5:**
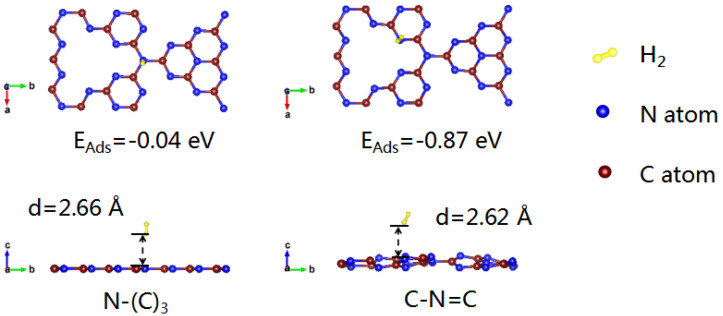
Adsorption energy of hydrogen on N-(C)_3_ and C-N=C.

In order to further confirm the selective deposition of Pt NPs on different types of nitrogen in different atmospheres, the adsorption energies of C-N=C and N-(C)_3_ to hydrogen were calculated using first-principles calculations. As shown in Figure 5, the adsorption energy of C-N=C for hydrogen (−0.87 eV) is much lower than that of N-(C)_3_ (−0.04 eV). Meanwhile, the adsorption of hydrogen at the C-N=C site causes obvious structural distortion of g-C_3_N_4_. As C-N=C is easier to adsorb hydrogen, more Pt NPs in CNH-Pt obtained by heat treatment in argon–hydrogen atmosphere will be adsorbed by N-(C)_3_. The adsorption capacity of C-N=C to Pt atoms is stronger than that of N-(C)_3_ [43], so Pt NPs in CNA-Pt obtained by air annealing without the influence of hydrogen are more likely to deposit on the position of C-N=C, which is consistent with XPS test results, confirming the hypothesis of selective deposition of Pt NPs.

**Figure 6:**
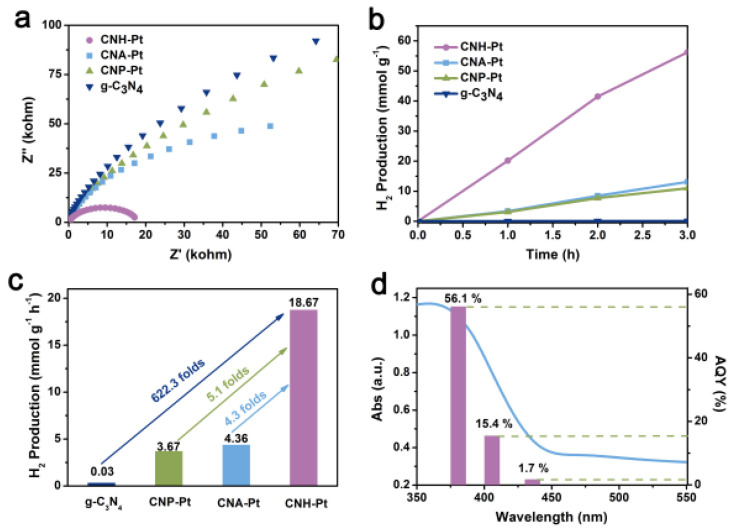
EIS Nyquist plots (**a**), time course of hydrogen evolution over 3 h (**b**) and hydrogen production rates (**c**) of CNH-Pt, CNA-Pt, CNP-Pt, and g-C_3_N_4_. (**d**) Wavelength-dependent AQY and absorption spectrum of CNH-Pt.

Electrochemical impedance spectroscopy (EIS) and photoluminescence (PL) was used to verify the electrical conductivity and the recombination rate of charge-carriers. Semicircular Nyquist plots of CNH-Pt show obviously reduced diameter (Figure 6a), indicating its smaller charge carrier transport resistance and higher separation efficiency of electrons and holes [42,44,45]. The PL peak of CNH-Pt at 470 nm is significantly lower than that of g-C_3_N_4_ (Figure S5), indicating more effective separation of photoexcited electrons and holes in CNH-Pt [46]. The results of EIS and PL show that Pt loading reduces the charge-carriers recombination rate of the material.

The photocatalytic hydrogen production performance of the catalyst was tested under full-spectrum simulated solar-light. Figure 6b shows the accumulation of hydrogen production over time within 3 h. The hydrogen production of pure g-C_3_N_4_ is very small, but the hydrogen production of the catalysts supported by Pt NPs all increases. Figure 6c shows the average hydrogen production rate of the catalysts, among which the average hydrogen production rate of CNH-Pt is up to 18.67 mmol·g^−1^·h^−1^, 622.3 times the 0.03 mmol·g^−1^·h^−1^ of pure g-C_3_N_4_. Comparing with pure g-C_3_N_4_, the average hydrogen production rates of CNP-Pt and CNA-Pt are also greatly improved, reaching 3.67 mmol·g^−1^·h^−1^ and 4.36 mmol·g^−1^·h^−1^, respectively, but far lower than that of CNH-Pt. The excellent photocatalytic performance of CNH-Pt may be attributed to the following two aspects: (i) Pt NPs with small size and high dispersion provide sufficient reaction sites for decomposition of aquatic hydrogen; (ii) The synergistic effect of Pt^0^ and Pt^2+^ promotes charge separation and transfer while inhibiting the unexpected hydrogen reverse reaction [42]. Cyclic stability test is shown in Figure S6, where the hydrogen production activity of CNH-Pt decreases with reaction time, possibly due to Pt leaching during the reaction. After 9 h of reaction, the samples were collected for ICP test, in which the Pt content was reduced to about 1 wt%. The wavelength-dependent apparent quantum yield (AQY) of CNH-Pt photocatalyst matches the light absorption spectra, and the AQY is estimated to be about 56.1% at 380 nm, 15.4% at 405 nm and 1.7% at 435 nm, respectively (Figure 6d). The AQY of CNH-Pt is better at 380 nm, which is consistent with its absorption capacity of light. The hydrogen production efficiency of CNH-Pt can reach 4.40 mmol·g^−1^·h^−1^, even under the very weak light intensity of 380 nm monochromatic light (obtained with 380 nm band pass filter) (Figure S7). Although CNH-Pt has slightly enhanced absorption of light above 400 nm compared with original g-C_3_N_4_, it still absorbs ultraviolet light mainly (Figure S8). Because ultraviolet light only accounts for about 5% of sunlight, the absorption spectrum of the sample should be broadened in the follow-up work to enhance the utilization of the solar slight.

Table 1 shows the main concerns and the photocatalytic activity of hydrogen production of previous works on the same topic. In the previous work, there is no study on the effect of selective deposition of Pt on the activity of photocatalytic hydrogen production, and the catalyst prepared in this work has a competitive advantage in the activity of hydrogen production, which can provide reference for the subsequent work in related fields.

## 4. Conclusions

In conclusion, in this paper, Pt NPs composed of Pt^0^ and Pt^2+^ were selectively deposited on tri-coordination graphitized nitrogen of g-C_3_N_4_ by heat-treatment in argon–hydrogen atmosphere, and thus photocatalyst with Pt cocatalyst of uniform size and good dispersity was achieved. From theoretical calculation, it is found that hydrogen is more easily adsorbed on pyridine nitrogen, so that more Pt NPs can be deposited on tri-coordination graphitized nitrogen. The internal mechanism of selective deposition of Pt NPs by heat-treatment in different atmospheres is clarified. The highly-dispersed Pt NPs promote the separation of electrons and holes and the transfer of electrons to hydrogen ion. The photocatalytic hydrogen productivity of this photocatalyst reaches 18.67 mmol·g^−1^·h^−1^, which is 4.3 times that of the photocatalyst when Pt aggregated nanoparticles are selectively deposited on pyridine nitrogen of g-C_3_N_4_, and 5.1 times that of the photocatalyst when Pt NPs of inhomogeneous size are indifferently deposited on g-C_3_N_4_. These results indicate that selective deposition of Pt cocatalyst on the surface of g-C_3_N_4_ will affect the size and distribution of Pt NPs, and then greatly influence the photocatalytic activity of the catalyst. The present work provides a reference for further study of anchoring noble metal cocatalyst on the semiconductor surface to improve the photocatalytic performance.

## Figures and Tables

**Table 1:** The main concerns and hydrogen evolution data of previous work.

Research Highlights	Pt Amount (wt%)	H_2_ Evolution Rate	Light Source	Sacrificial Agent	Ref.
Effect of Pt Shape	1	588 µmol g^−1^ h^−1^	350 W Xe lamp (>400 nm)	10% TEOA	[25]
Order of addition of sacrificial agent	1	4210 µmol g^−1^ h^−1^	350 W Xe lamp	10% TEOA	[27]
Reactive metal-support interaction to stabilize Pt single atoms	1.72	3020 µmol g^−1^ h^−1^	300 W Xe lamp (>420 nm)	10% TEOA	[36]
A bridging Pt-N bond boosted electron transfer	1.24	11,472 µmol g^−1^ h^−1^	300 W Xe lamp	20% TEOA	[37]
Effect of Pt size	3	473.82 µmol mg^−1^ Pt for 4 h	>420 nm	10% methanol	[38]
Anchoring Pt^2+^/Pt^0^ on g-C_3_N_4_ nitrogen vacancies	0.5	2626 µmol g^−1^ h^−1^	300 W Xe lamp (>420 nm)	15% TEOA	[42]
Selective deposition of Pt	3	18,670 µmol g^−1^ h^−1^	300 W Xe lamp (AM1.5)	25% TEOA	This work

## Data Availability

The data presented in this study are available on request from the corresponding author.

## Supplementary Materials

The following are available online at https://www.mdpi.com/article/10.3390/nano11123266/s1, Table S1: The theoretical and actual loading Pt (wt%) of catalyst; Figure S1. XRD patterns of CNH, CNA and g-C_3_N_4_; Figure S2. XPS spectra of N 1s of catalysts with three functional groups; Figure S3. XPS spectra of C 1s of CNH-Pt and CNH (a), CNA-Pt and CNA (b), CNP-Pt and g-C_3_N_4_ (c); Figure S4. The ratio of N-(C)_3_, C-N=C and C-N-H before and after Pt loading on the catalysts; Figure S5. PL spectra of g-C_3_N_4_ and CNH-Pt; Figure S6 Recycle ability of CNH-Pt for the photocatalytic H_2_ evolution; Figure S7. Time course of hydrogen evolution over 3 h of CNH-Pt under 380 nm monochromatic light (a), the transmission spectrum of the 380nm band-pass filter (b); Figure S8. UV-vis absorption spectra of CNH-Pt, CNA-Pt, CNP-Pt and g-C_3_N_4_.
