# Supplementary material for: Enhancing Photocatalytic Hydrogen Production of g-C3N4 by Selective Deposition of Pt Cocatalyst
Source: Nanomaterials (Basel). 2021 Nov 30;11(12):3266. doi: 10.3390/nano11123266 (PMC8709060; doi:10.3390/nano11123266)
Supplement: Supplementary file 1 [file nanomaterials-11-03266-s001.zip › nanomaterials-1458689 supplementary.pdf]

nanomaterials

Supplementary Material

Enhancing Photocatalytic Hydrogen Production of g-C_3N_4 by Selective Deposition of Pt Cocatalyst

Yang Li [1], Yue Lu [1], Zhaoyu Ma [1], Lianqing Dong [2], Xiaofang Jia [1] and Junying Zhang [1,*]

[1] School of Physics, Beihang University, Beijing 100191, China; liyang1619125@buaa.edu.cn (Yang Li); Luyue@buaa.edu.cn (Yue Lu); 18338208022@163.com (Z.M.); jiaxiaofang@buaa.edu.cn (X.J.)
[2] Beijing Institute of Space Mechanics & Electricity, Beijing 100094, China; XPHDLQ@163.com
* Correspondence: zjy@buaa.edu.cn

1. Characterization

X-ray powder diffraction (XRD) patterns were characterized using Bruker D8 Advacend X-ray diffractometer (Karlsruhe, Germany). The surface morphology and structure of the samples were recorded on transmission electron microscopy (TEM, JEM-2100F, JEOL, Akishima-shi, Japan). UV–vis diffuse reflectance spectra (DRS) were obtained by HITACHI U-3010 UV–vis spectrophotometer (Tokyo, Japan). X-ray photoelectron spectroscopy (XPS) was measured on Thermo Scientific ESCALAB 250Xi (Massachusetts, USA). The content of Pt elements in the samples was detected by an Inductively Coupled Plasma Optical Emission Spectrometer (ICP-OES) on Agilent ICPOES730 (Palo Alto, USA). Luminescence spectra were recorded on FLS 980 fluorescence spectrometer (Edinburgh, UK).

2. Photocatalytic and Photoelectrochemical Measurements

The photocatalytic hydrogen production activity of the materials was tested under simulated solar light irradiation. Typically, 10 mg sample was dispersed in 40 mL 25% of triethanolamine solution (triethanolamine as the sacrificial agent). To remove air completely, high purity argon was injected into the reaction solution for 10 min before the reaction. The light source was a 300 W (Perfectlight, Beijing, China) xenon lamp with an AM 1.5 filter. During the reaction, the solution was kept at about 10 °C through the circulating cooling water system (Beijing Changliu, Beijing, China). The products gas was detected by gas chromatography (FULI GC9790 PLUS, Taizhou, China) using argon as carrier gas with a thermal conductivity detector (TCD). The apparent quantum yield (AQY) was tested by a 300 W Xe lamp (Perfectlight, Beijing, China) with 380, 405, and 435 nm band-pass filter. The calculation formula of AQY is: AQY = (2 × number of produced H_2/number of absorbed photons) × 100%. The photoelectrochemical measurements were made at the CHI604C electrochemical work station (Shanghai Chenhua, Shanghai, China). A standard three-electrode system using Pt as counter electrode, photocatalyst electrode coated on glassy carbon electrode as working electrode, and Ag/AgCl (in saturated KCl) as reference electrode was adopted. 0.2 M Na_2SO_3 was used as the electrolyte. The working electrodes were prepared as follows: 10 mg catalyst was dissolved in 0.1 ml alcohol and 0.1 ml nafion. After 20 min of ultrasonic treatment, 0.01 ml mixture was dropped on the glassy carbon electrode and dried naturally.

3. Density Functional Theory Calculation Method

The calculation was performed using density functional theory (DFT) and the projection enhancement wave (PAW) pseudo-pulse implemented in the Ab initio Simulation Package (VASP) in Vienna. We used the perdew-burke-ernzerhof (PBE) generalized gradient approximation (GGA) to describe the exchange potential and the associated potential [1,2]. In addition, the empirical correction method PBE + D2 (D stands for dispersion) was used to describe the VDW interaction [3]. The energy cutoff of plane wave expansion

was 500 eV. In order to obtain the optimized geometry, the energy convergence criterion 1.0×10^{-4} eV and the force convergence tolerance <0.02 eV/Å were used. The interaction between adjacent plates was avoided by using a vacuum region of 15 Å in the Z direction. For the g-C_3N_4 monolayer, 3 × 2 × 1 k point grid was used for geometric optimization. The adsorption energy (E_{ads}) between a slab and adsorbed gas molecules was computed as

$$E_{ads} = E_{molecule+C3N4} - E_{C3N4} - E_{molecule} \quad (1)$$

where, E_{C3N4} and $E_{molecule+C3N4}$ is the total energies of the system before and after the adsorption of gas molecules, respectively. $E_{molecule}$ is the energy of a gas molecule.

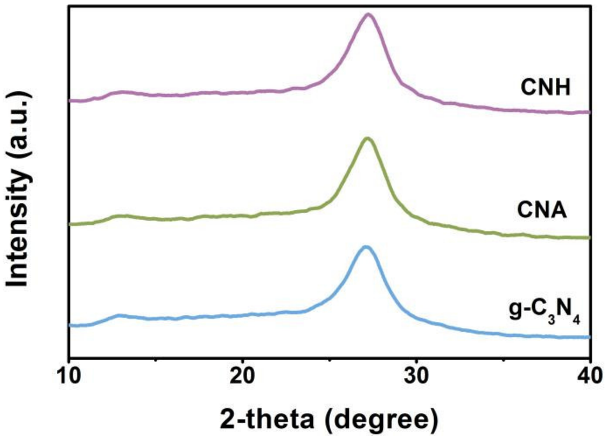

Figure S1. XRD patterns of CNH, CNA and g-C_3N_4.

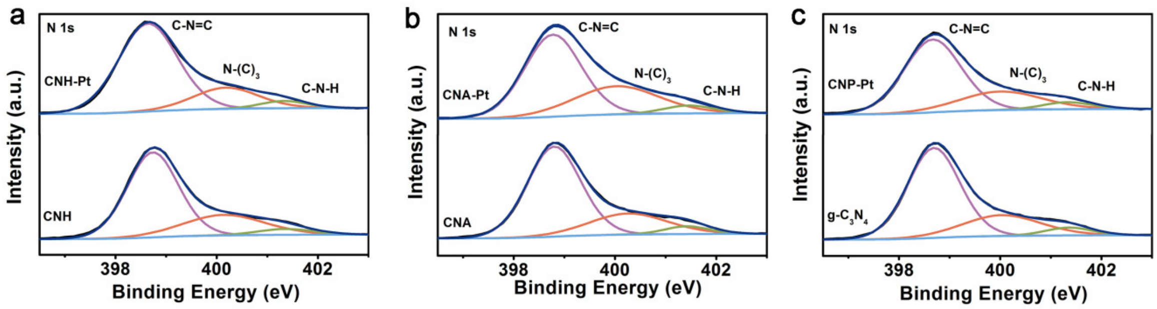

Figure S2. XPS spectra of N 1s of catalysts with three functional groups (**a**–**c**).

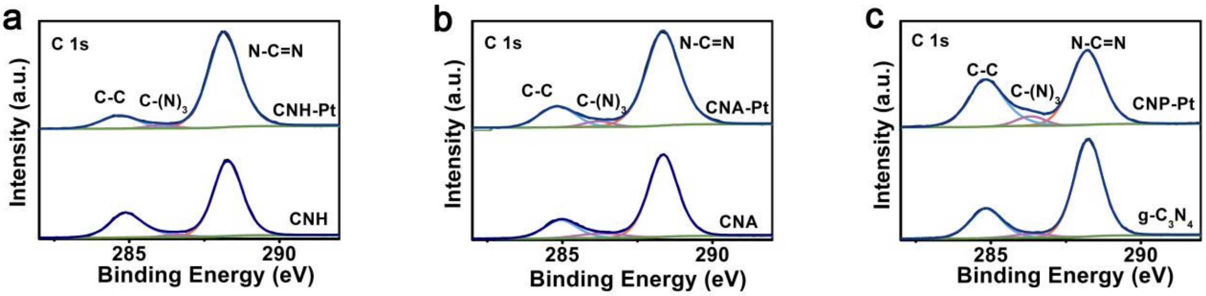

Figure S3. XPS spectra of C 1s of CNH-Pt and CNH (**a**), CNA-Pt and CNA (**b**), CNP-Pt and g-C_3N_4 (**c**).

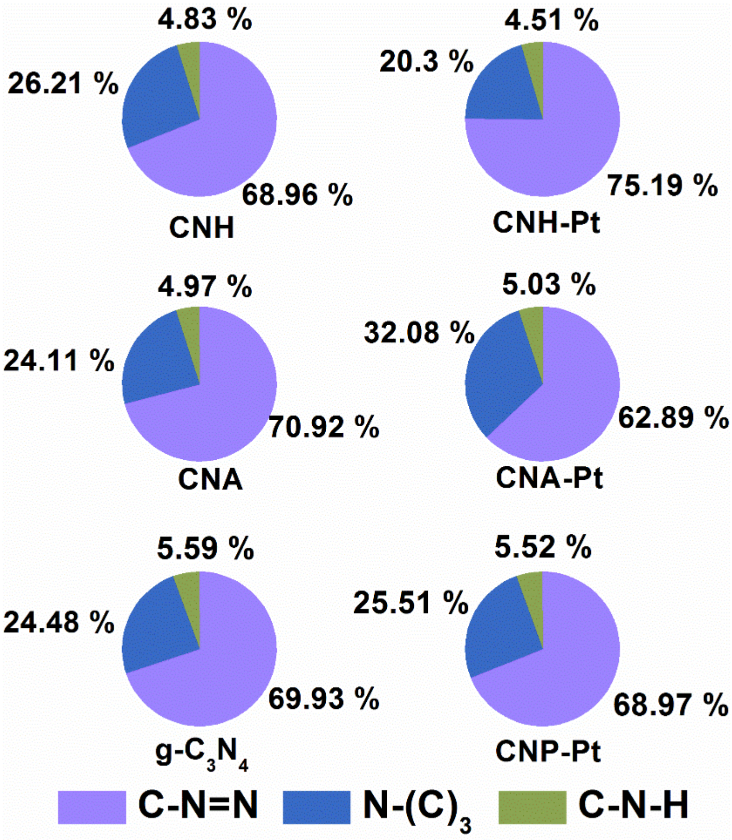

Figure S4. The ratio of N-(C)$_3$, C-N=C and C-N-H before and after Pt loading on the catalysts.

Table S1. The nominal and actual loading Pt (wt%) of the catalysts.

Catalyst	Nominal loading Pt (wt%)	Actual loading Pt (wt%)
CNH-Pt	3	2.8
CNA-Pt	3	3.5
CNP-Pt	3	2.9

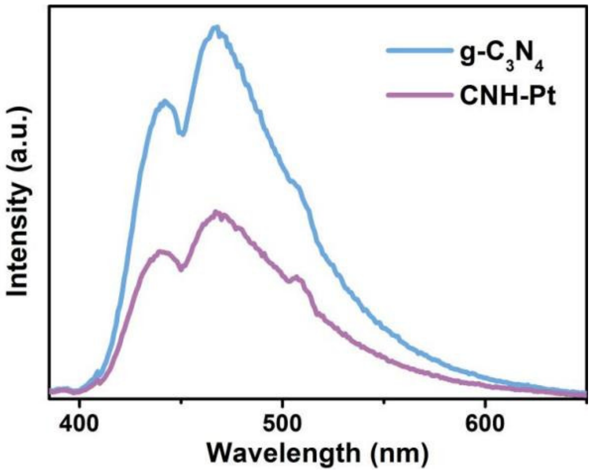

Figure S5. PL spectra of g-C_3N_4 and CNH-Pt.

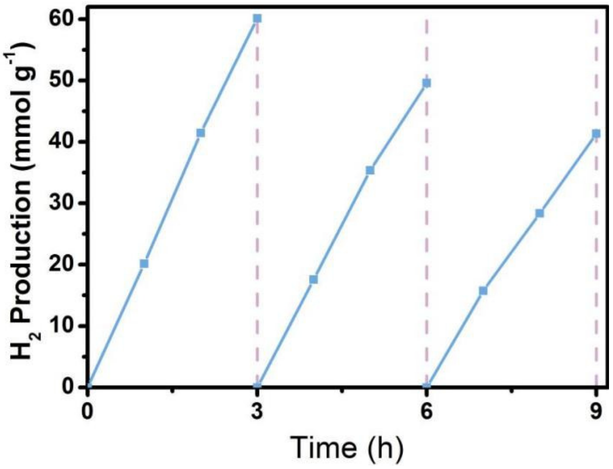

Figure S6. Recycle ability of CNH-Pt for the photocatalytic H_2 evolution.

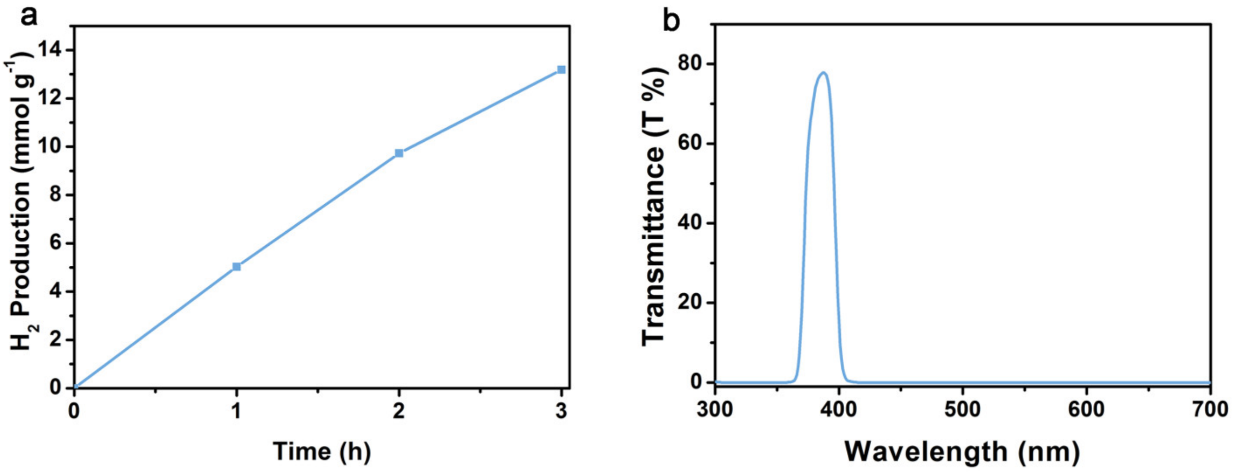

Figure S7. Time course of hydrogen evolution over 3 h of CNH-Pt under 380 nm monochromatic light (**a**), the transmission spectrum of the 380 nm band-pass filter (**b**).

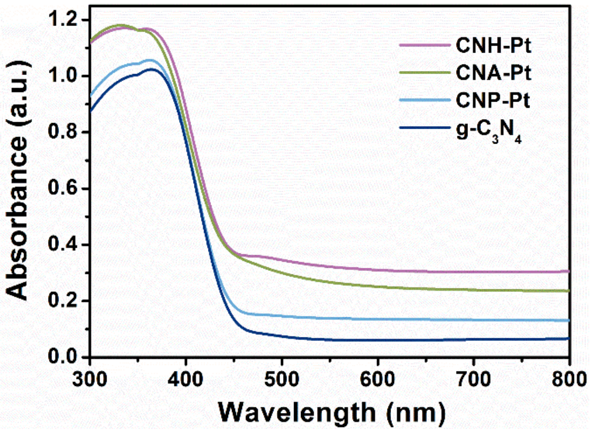

Figure S8. UV-vis absorption spectra of CNH-Pt, CNA-Pt, CNP-Pt and g-C_3N_4.

References

[1] Kresse, G.; Furthmüller, J. Efficient Iterative Schemes for Ab Initio Total-Energy Calculations Using a Plane-Wave Basis Set, *Phys. Rev. B* **1996**, *54* 11169.

[2] Kresse, G.; Joubert, D. From ultrasoft pseudopotentials to the projector augmented-wave method, *Phys. Rev. B* **1999**, *59* 1758.

[3] Grimme, S. Semiempirical GGA-type density functional constructed with a long-range dispersion correction, *Journal of computational chemistry* **2006**, *27*, 1787–1799.
